# 

**Published:** 1881-07

**Authors:** 


JULY, 1881.

TWENTY-EIGHTH YEAR.

### HALL'S JOURNAL OF HEALTH

*E. H. GIBBS, A.M., M.D., Editor,*

No. 141 EIGHTH STREET (Near Broadway), NEW YORK.

TERMS: $2 a Year, or $1 for Six Months. Single number, 20 cts.

